# Influence of Technological Factors on the Quality of Chitosan Microcapsules with *Boswellia serata* L. Essential Oil

**DOI:** 10.3390/pharmaceutics14061259

**Published:** 2022-06-13

**Authors:** Lauryna Pudziuvelyte, Aiste Siauruseviciute, Ramune Morkuniene, Robertas Lazauskas, Jurga Bernatoniene

**Affiliations:** 1 Institute of Pharmaceutical Technologies, Medical Academy, Lithuanian University of Health Sciences, Sukileliu pr. 13, LT-50161 Kaunas, Lithuania; lauryna.pudziuvelyte@lsmuni.lt; 2 Department of Drug Technology and Social Pharmacy, Lithuanian University of Health Sciences, Eiveniu 4, LT-50161 Kaunas, Lithuania; aiste.siauruseviciute@stud.lsmuni.lt; 3 Department of Drug Chemistry, Lithuanian University of Health Sciences, Eiveniu 4, LT-50161 Kaunas, Lithuania; ramune.morkuniene@lsmuni.lt; 4 Institute of Physiology and Pharmacology, Lithuanian University of Health Sciences, A. Mickeviciaus 7, LT-44307 Kaunas, Lithuania; robertas.lazauskas@lsmuni.lt

**Keywords:** chitosan, microcapsules, essential oil, *Boswellia serata*

## Abstract

Essential oils contain many volatile compounds that are not stable and lose their pharmacological effect when exposed to the environment. The aim of this study is to protect *Boswellia serrata* L. essential oil from environmental factors by encapsulation and determine the influence of chitosan concentration and types (2%, 4%; medium and high molecular weights), essential oil concentration, different emulsifiers (Tween and Span), and technological factors (stirring time, launch height, drip rate) on the physical parameters, morphology, texture, and other parameters of the generated gels, emulsions, and microcapsules. For the first time, *Boswellia serrata* L. essential oil microcapsules with chitosan were prepared by coacervation. Hardness, consistency, stickiness, viscosity, and pH of chitosan gels were tested. Freshly obtained microcapsules were examined for moisture, hardness, resistance to compression, size, and morphology. Results show that different molecular weights and concentrations of chitosan affected gel hardness, consistency, stickiness, viscosity, mobility, and adhesion. An increase in chitosan concentration from 2% to 4% significantly changed the appearance of the microcapsules. It was found that spherical microcapsules were formed when using MMW and HMW 80/1000 chitosan. Chitosan molecular weight, concentration, essential oil concentration, and stirring time all had an impact on the hardness of the microcapsules and their resistance to compression.

## 1. Introduction

Essential oils are a complex mixture of compounds that are sensitive to oxygen, light, and high temperatures [1,2]. These environmental factors contribute to the degradation of the active substances and a reduction in the biological effects of the essential oil [2]. Microencapsulation technology is used to preserve the properties of an essential oil, during which it is coated with a protective shell [3,4]. Microencapsulated essential oils have a wide variety of applications across a range of food, cosmetic, and pharmaceutical products [5]. Microencapsulation of essential oils ensures the stability of volatile compounds during thermal processing, as well as during conversion of liquids to powder, and results in a slow and controlled release of the active compounds [5,6]. Various materials, such as soluble polymers, micro/nanocapsules, liposomes, micelles, and emulsions, have been developed and formulated as effective microencapsulation carriers [7]. Coacervation is one of the most efficient and frequently used microencapsulation methods in the pharmaceutical industry [8]. Coacervation-based microencapsulation is suitable for lipophilic materials, such as essential oils, vegetable oils and resins, and vitamin E, however the process has also potential for the encapsulation of hydrophilic substances [9]. This method involves a liquid-liquid phase separation with coacervate formation between positively charged polymers, usually proteins or polysaccharides [10]. Proteins can be divided into two types, animal or plant. Examples of animal proteins include gelatin, whey protein, and albumin. Plant proteins include soy, pea, and cereal. Examples of polymeric polysaccharides are gum arabic, pectin, chitosan, agar, alginate, and others [11]. Coacervation is superior to other microencapsulation techniques because of its high loading capacity, low temperature, reduced evaporation losses or thermal degradation, and compatibility with controlled release of the active substances. There is no need to use specific equipment for its implementation and it has simple preparation conditions, such as non-toxic solvents and low agitation utilization [12]. The main disadvantages of this technique are that it is challenging to scale up and the possibility of agglomerate formation [13].

Over the last thirty years, pH-sensitive hydrogels based on marine biopolymers have gained immense interest for microencapsulation of active ingredients. The interest in marine biopolymers is due to their beneficial features, such as low-cost production, excellent biocompatibility, tunable biodegradability, and non-toxicity [7,14]. Chitosan is a natural polysaccharide composed of glucosamine and N-acetylglucosamine that are linked by beta-1,4 glycosidic linkages [14,15]. Chitosan is not found in nature but is obtained from chitin by deacetylation [14,16]. Chitin is extracted from the skeletons of crustaceans and insects (primary sources for chitin extraction) and the cell walls of fungi [10,17]. It has a strong crystalline structure due to the hydrogen interaction between the acetamide and hydroxyl groups. Chitin is not easily adapted due to the high level and strong structure of the acetylated groups, as well as its poor solubility in aqueous solutions. Partial deacetylation of chitin and its conversion to chitosan increases the content of amino groups and its water solubility [18]. The average molecular weight of chitosan ranges from 50 to 2000 kDa [19], which affects its viscosity, solubility, and elasticity. In an alkaline or neutral environment, the free amino groups of chitosan are not protonated, and therefore, are insoluble in water. At acidic pH, these groups are protonated, the polysaccharide is positively charged and soluble in water [16]. Chitosan is a pseudoplastic substance that increases in viscosity very well in an acidic environment. The viscosity of chitosan solutions depends on temperature and concentration [19,20]. Chitosan is characterized by the following activities: antibacterial, antifungal, analgesic, mucoadhesive and hemostatic [14,19,21].

*Boswellia serrata* L. Roxb. ex Colebr. is a plant belonging to the family *Burseraceae* [22]. It is a deciduous tree growing up to 18 m in height and expanding to 2.4 m in width (usually, 1.5 m). Boswellia serrata grows in the humid and mountainous regions of India, North Africa, and the Middle East [22,23]. The essential oil of Boswellia serrata consists of a mixture of monoterpenes, diterpenes, and sesquiterpenes [24]. One study showed that 29 components were present in the essential oil of Boswellia serrata harvested in North India, including 3-carene (34.74%), β-ocimene (13.78%), d-limonene (8.25%), β-caryophyllene (6.65%), and terpinolene (5.39%) [25]. Boswellia essential oil has antimicrobial, antioxidant, and anti-inflammatory activities [24,25,26].

In the present study, the formation of stable microcapsules with chitosan containing *Boswellia serrata* essential oil is reported for the first time. Different types of chitosan were used to compare their ability to form gels and microcapsules. The production of microcapsules from the chitosan gels and emulsions was achieved by cross-linking with sodium hydroxide (complex coacervation). The influence of technological factors (chitosan type, concentration of emulsifiers and *Boswellia serrata* essential oil, stirring time, drip rate, and others) on the quality of the chitosan gels and microcapsules was evaluated (pH value, hardness, consistency, moisture content, stickiness, viscosity, mobility, and adhesion). The properties and characteristics of the microcapsules were examined by microscopy.

## 2. Materials and Methods

### 2.1. Materials

Medium molecular weight (MMW) chitosan, high molecular weight (HMW) 80/1000 chitosan, and high molecular weight (HMW) 80/3000 chitosan were purchased from Sigma-Aldrich (Steinheim, Germany). Essential oil obtained from *Boswellia serrata* resin was obtained from “Kvapų namai”, Kaunas, Lithuania. Emulsifiers Tween 20^®^, Tween 80^®^, Span 20^®^, and Span 80^®^ were purchased from Sigma-Aldrich (Germany). Acetic acid (100%) and sodium hydroxide were obtained from Sigma-Aldrich (Germany). Purified water for experiments was produced with a Millipore Super Purity Water System (Sigma-Aldrich Corp., St. Louis, MO, USA).

### 2.2. Production of Chitosan Gels

Chitosan gels of different concentrations and molecular weights were produced: 2% MMW chitosan gel, 4% MMW chitosan gel, 2% HMW 80/1000 chitosan gel, and 2% HMW 80/3000 chitosan gel. For gel production, the required amount of chitosan powder was weighed and slowly added to a 3% acetic acid solution and stirred using a IKA EUROSTAR 200 digital stirrer set at 500 revolutions per minute (rpm) until a clear gel was formed. Since a large amount of air bubbles formed in the gel during stirring, the prepared gel was transferred to 50 mL centrifuge tubes and centrifuged for 2 min at 2000 rpm speed in a Sigma 3–18KS centrifuge to remove air bubbles from the gel.

### 2.3. Determination of pH Values of Chitosan Gels

Aqueous gel solutions containing 5% chitosan were prepared from chitosan with different molecular weights, and the pH was measured by using a WinLab^®^ Data Line pH-Meter [27]. Initially, the pH-meter electrode was calibrated in a beaker with purified water until a constant pH value was reached, then the pH of a 5% aqueous chitosan gel solution was measured. Each solution was measured three times, and the pH meter electrode was calibrated in purified water before each new measurement.

### 2.4. Texture Analysis of Chitosan Gels

A texture analysis of the chitosan gels was prepared according Ferreira et al. [28] with some modifications. A texture analyzer TA.XTplus (Stable Micro Systems, Godalming, UK) was used. Each gel was poured into a container and placed on the stage of the texture analyzer. The required tap A/BE was attached to the rod of the appliance, the size of which corresponded to the size of the container. Initially, a backward extrusion test was performed. The required T.A. parameters—15 mm distance and 10 mm/s speed—were selected in the settings. The test measured the hardness (g), consistency (g*s), stickiness (g), and viscosity index (g*s) of the gel. Each sample was analyzed three times, and the program automatically created a graph of the results.

A spreadability test was also performed with the same T.A. settings (15 mm distance and 10 mm/s speed). In the data processing section of the program, the calculations for the peak negative force and negative area were selected. This test measured mobility (g*s) and adhesion (g*s). The sample was analyzed three times, and the program created a graph of the results.

### 2.5. Formation of Microcapsules from Chitosan Gels

Microcapsules from chitosan gels were formatted according to the method of Souza et al. [29] with some modifications. The reconstituted chitosan gel was placed into a 10 mL syringe with a needle attached. A 1% NaOH solution was poured into a beaker, which was placed on MSH-20A magnetic stirrer. The mixing speed of the magnetic stirrer was set at 130 rpm. Chitosan gel was slowly added to the 1% NaOH solution. The microcapsules were formed with previously prepared chitosan gels (2% MMW, 4% MMW, 2% HMW 80/1000, 2% HMW 80/3000) dripped from different launch heights—4 and 10 cm—from the point of droplet release to the surface of the 1% NaOH solution in the beaker using different stirring times: 5, 15, and 30 min (Table 1). The microcapsules were rinsed several times with purified water, placed on filter paper, and left to dry for 24 h.

### 2.6. Gas Chromatography-Mass Spectrometry (GC-MS) Analysis of Boswellia serrata Essential Oil 

This assay was performed using a headspace technique on a Shimadzu GC-MS-QP2010 gas chromatograph-mass spectrometer system (Shimadzu, Tokyo, Japan) equipped with a Shimadzu autoinjector AOG-5000 (Shimadzu, Tokyo, Japan) with an Rxi^®^-5 ms capillary column (length 30 m, diameter 0.25 mm, stationary phase layer thickness 0.25 μm) according to Pudziuvelyte et al. [30]. The temperature in the column was set at 50 °C for 5 min, then raised 2 °C/min up to 200 °C, then 15 °C/min up to 315 °C, and this temperature was maintained constant for 15 min. Injector temperature was 260 °C. Helium gas was used for the analysis. The sample was injected using a flow ratio of 1:60. The injection volume was 1 μL. Mass range 29–500 amu, scan time 0.2 s, injection site temperature 280 °C.

### 2.7. Preparation of Emulsions

The selected amount of chitosan gel was placed in a beaker, the required amount of emulsifier was added dropwise, and everything was mixed. Then the required amount of *Boswellia serrata* essential oil was added and mixed (Table 2).

### 2.8. Microscopic Analysis of Emulsions

Microscopy was used to assess the size of the essential oil droplets in the emulsion and their distribution [27]. One drop of emulsion was placed on a slide and covered with a coverslip. A Motic^®^ BA310 microscope with a Nis-Elements program was used to evaluate the image. The images were magnified 100 times and the program calculated the size of selected oil droplets. For each emulsion, 10 random droplets of oil were selected, and their average diameter was determined.

### 2.9. Emulsions Stability Study by Centrifugation

The prepared emulsions were placed in tubes and centrifuged by using a Sigma 3-18KS centrifuge. The spin time was set for 5 min at 10,000 rpm [27]. After centrifugation, the oil-phase and aqueous phase of the emulsions were separated.

### 2.10. Formation of Microcapsules with a Syringe Pump

**Figure 1:**
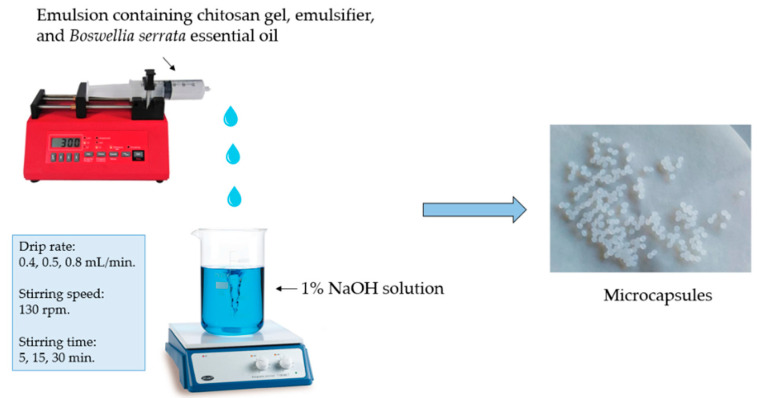
Scheme of microcapsules formation from emulsion.

The selected chitosan gel or emulsion was drawn into a 10 mL medical syringe with a needle attached. The syringe was attached to a LA-120 syringe pump (Jiangsu Zhengkang Medical Apparatus, Shanghai-Nanjing Railway, Changzhou, China) [31]. The gel was dripped at a rate of 0.5 mL/min, and the emulsion at a rate of 0.4 mL/min or 0.8 mL/min, with a diameter of 14.43, depending on the volume of the syringe (Figure 1).

A beaker with 1% NaOH solution was placed on a MSH-20A magnetic stirrer set at 130 rpm stirring speed. The chitosan gel or emulsion was added to the 1% NaOH solution from a height of 10 cm (from the starting point of the gel drop to the surface of 1% NaOH solution). The microcapsules were produced using different stirring times: 5, 15, and 30 min. (Table 3). Freshly made microcapsules were rinsed several times with purified water and placed on filter paper to dry for 24 h.

### 2.11. Moisture Content

A moisture analyzer (Kern MLS, Balingen, Germany) was used to measure moisture content. The sample (0.1–0.2 g) was dried at 100–105 °C until a constant weight was obtained [32]. Samples were tested 2, 4, 6, and 24 h after preparation of the microcapsules. The test was repeated three times and the average of the obtained results was derived.

### 2.12. Compression Analysis

A Stable Micro Systems manual TA.XTplus texture analyzer was used to measure the crush resistance of fresh microcapsule shells. A pre-programed gnocchi compression test was chosen for this purpose. Ten fresh microcapsules from each series were placed on the surface of the texture analyzer, a flat base was attached to the device, and the maximum force of the device was set to 6500 g [31]. Other parameters were selected in the program settings—the descent distance to microcapsules, 3 mm, and the speed of descent, 2 mm/s. The base descended, pressed the microcapsules, then returned to its starting position. The test was repeated three times for each series, and the program automatically created a graph of the results.

### 2.13. Microscopic Analysis of Microcapsules

To assess the appearance of the dried microcapsules and their size, a microscopic examination was performed using a Motic^®^ BA310 microscope with the Nis-Elements program [33]. The microcapsules in each series were placed on a slide and the image was magnified 100 times. Three microcapsules were randomly selected from each series.

### 2.14. Statistical Analysis

Data obtained during the research were processed by Microsoft Office Excel 2016 (Redmond, WA, USA). The results were summarized, and their means and standard deviations were calculated. Student’s *t*-test was used to assess the differences in results. Differences were considered statistically significant when *p* < 0.05.

## 3. Results and Discussion

### 3.1. Influence of Chitosan Molecular Weight and Concentration on Gel pH Values

Gels were prepared from chitosan with different molecular weights and concentrations and their pH values were determined. The obtained results are shown in Table 4.

We compared 2% and 4% chitosan gels with different molecular weights. The pH values ranged from 3.34 ± 0.05 to 3.65 ± 0.03. When comparing different molecular weight chitosan gels at the same concentration, the changes in pH were insignificant. The lowest pH value was observed for 2% HMW 80/1000 chitosan gel (3.34 ± 0.05), which was slightly higher for the 2% HMW 80/3000 (3.35 ± 0.04) and 2% MMW (3.37 ± 0.03) chitosan gels. A significant difference was found for the 4% MMW chitosan gel, which had a maximum pH of 3.65 ± 0.03.

MMW chitosan was used in previous study, where it was dissolved in a 1% aqueous solution of lactic acid. The pH values ranged from 4.8 to 5.27. Compared to the results of our study, these values are higher due to the lower concentration of acid. Since this study aimed to produce a gel for application on the skin, these pH values were close to the pH value of skin (about 5). An acidic environment of the gel prevents the multiplication of microorganisms [34].

Senyigit et al. [35] used chitosan with small, medium, and large molecular weights dissolved in a 1% lactic acid solution, and measured pH values ranging from 4.98 ± 0.03 to 5.34 ± 0.04. The molecular weight of chitosan had no significant effect on pH.

Summarizing the obtained results, it can be stated that the molecular weight of chitosan did not affect the pH value of the gels, but the concentration of chitosan did. A higher concentration of chitosan had a significantly higher pH value (*p* < 0.05).

### 3.2. Influence of Different Molecular Weights and Concentrations of Chitosan on Gel Texture

**Figure 2:**
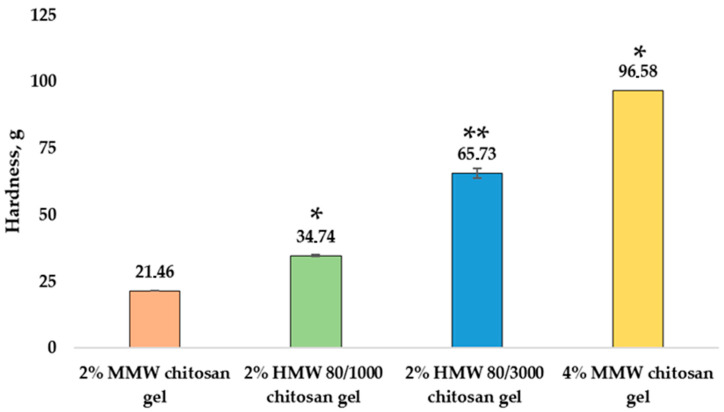
Hardness of chitosan gels. * *p* < 0.05 vs. MMW chitosan gel, 2%, ** *p* < 0.05 vs. HMW 80/1000 chitosan gel, 2%; *n* = 3.

Hardness (g), consistency (g*s), stickiness (g) and viscosity index (g*s) results were obtained by analyzing the texture of chitosan gels with different molecular weights and concentrations during the backward extrusion test. The hardness (g) results of the gels are shown in Figure 2.

A significant difference was found for the hardness of chitosan gels with different molecular weights. The 2% MMW chitosan gel had the lowest hardness, 21.46 ± 0.03 g; the 2% HMW 80/1000 chitosan gel had a medium average hardness, 34.74 ± 0.21 g; and the 2% HMW 80/3000 chitosan gel had highest hardness, 65.73 ± 1.79 g. A significant difference was found when comparing MMW chitosan gels of different concentrations: the 4% MMW chitosan gel was 4.5 times harder than the 2% chitosan gel. Sezer et al. [36] found that the hardness of their gel formulations were significantly affected by the molecular weight and concentration of the polymer. The hardness value of their hydrogel increased significantly 5-fold when the chitosan concentration was increased in from 1.5 to 2% [36].

**Figure 3:**
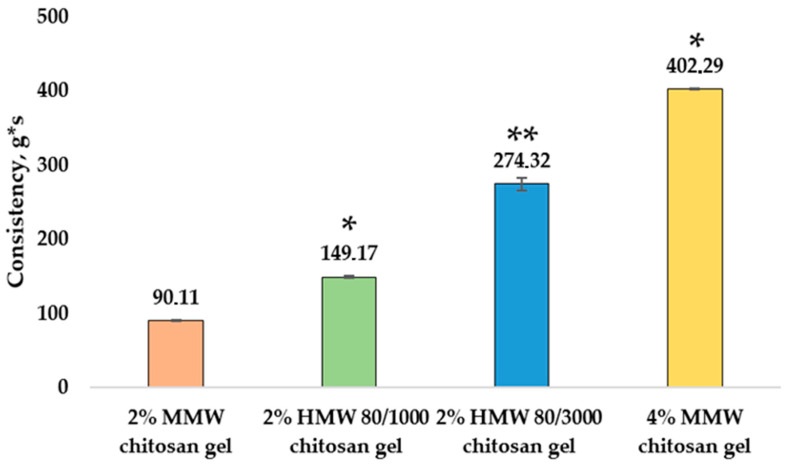
Consistency of chitosan gels. * *p* < 0.05 vs. MMW chitosan gel, 2%, ** *p* < 0.05 vs. HMW 80/1000 chitosan gel, 2%; *n* = 3.

After determining the consistency (g*s) of chitosan with different molecular weights and concentrations, a significant difference was found. Figure 3 shows the consistency (g*s) results of the gels. 

Results show the impact of chitosan concentration and molecular weight on gel consistency. When evaluating the consistency of chitosan gels with different molecular weights at the same concentration, the 2% MMW chitosan gel had the lowest value (90.11 ± 0.92 g*s), followed by the 2% HMW 80/1000 chitosan gel (149.17 ± 0.72 g*s), and the 2% HMW 80/3000 chitosan gel (274.32 ± 8.66 g*s). The 4% MMW chitosan gel had a significantly higher consistency (402.29 ± 0.62 g*s) than the 2% MMW chitosan gel. According to Szczesniak et al. [37], higher concentrations of chitosan increased the consistency of the gels, where the consistency of a 5% chitosan gel was higher than 1–4% chitosan gels.

**Figure 4:**
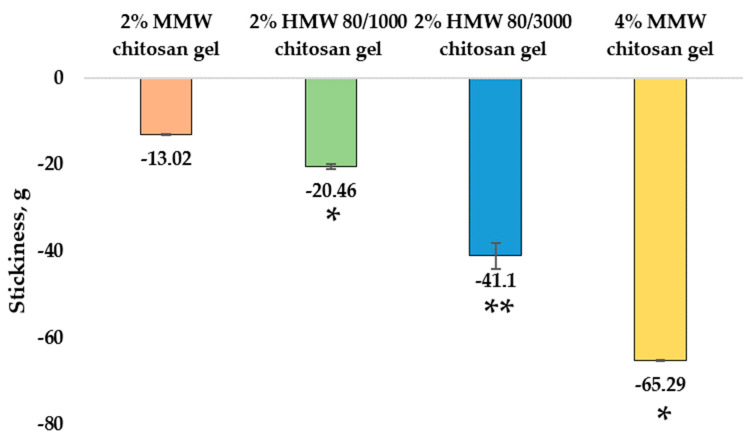
Stickiness of chitosan gels. * *p* < 0.05 vs. MMW chitosan gel, 2%, ** *p* < 0.05 vs HMW 80/1000 chitosan gel, 2%; *n* = 3.

The stickiness (g) of the gels was found to vary from −13.02 ± 0.13 g to −65.29 ± 0.11 g with significant differences. The obtained results are shown in Figure 4. Among the chitosan gels with different molecular weights at the same concentration, the least sticky was 2% MMW chitosan gel (−13.02 ± 0.13 g), and the most was 2% HMW 80/3000 chitosan gel (−41.10 ± 2.99 g), with the 2% HMW 80/1000 chitosan gel (−20.46 ± 0.66 g) in between. The 4% MMW chitosan gel had significantly higher stickiness (65.29 ± 0.11 g) than the 2% MMW chitosan gel.

**Figure 5:**
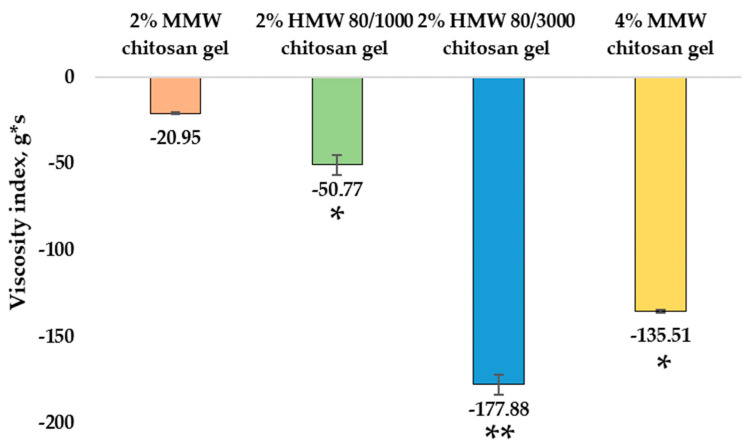
Viscosity index results of chitosan gels. * *p* < 0.05 vs. MMW chitosan gel, 2%, ** *p* < 0.05 vs. HMW 80/1000 chitosan gel, 2%; *n* = 3.

The viscosity indices ranged from −20.95 ± 0.39 g*s to −177.88 ± 5.79 g*s. Among the chitosan gels with the same concentration but different molecular weights, the 2% MMW chitosan gel had lowest viscosity index (−20.95 ± 0.39 g*s), followed by the 2% HMW 80/1000 chitosan gel (−50.77 ± 5, 74 g*s) and the 2% HMW 80/3000 chitosan gel (−177.88 ± 5.79 g*s). The difference between these results is significant. When evaluating gels with different concentrations, the 4% MMW chitosan gel had a significantly higher viscosity index (−135.51 ± 0.64 g*s) than the 2% MMW chitosan gel. The results are shown in Figure 5.

**Figure 6:**
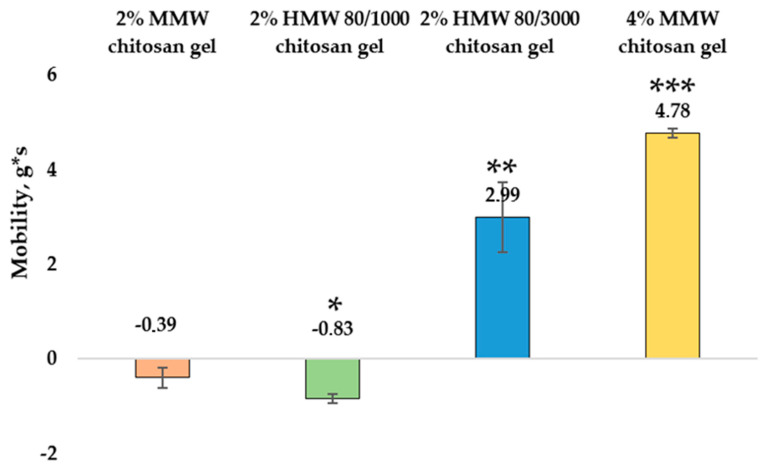
Mobility of chitosan gels. * *p* > 0.05 vs. MMW chitosan gel, 2%, ** *p* < 0.05 vs. HMW 80/1000 chitosan gel, 2%, *** *p* > 0.05 vs. MMW chitosan gel, 2%; *n* = 3.

The spreadability test was performed to determine the mobility (g*s) and adhesion (g*s) of the chitosan gels. The mobility values for chitosan gels with different molecular weights were found to range from −0.39 ± 0.22 g*s to 2.99 ± 0.74 g*s. The mobility (g*s) results for the gels are shown in Figure 6.

**Figure 7:**
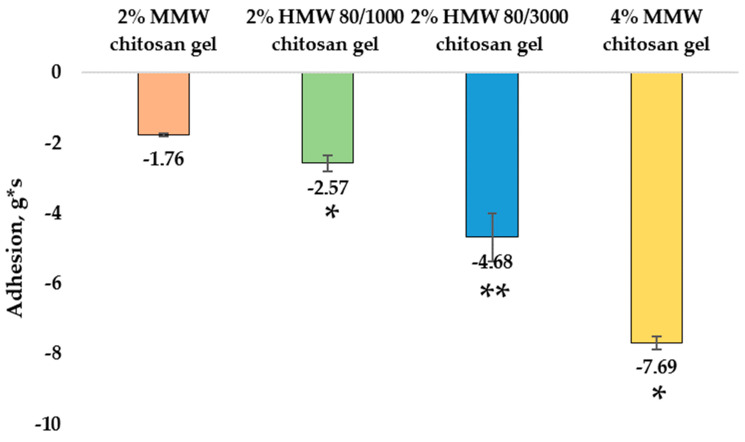
Adhesion of chitosan gels. * *p* < 0.05 vs. MMW chitosan gel, 2%, ** *p* < 0.05 vs. HMW 80/1000 chitosan gel, 2%; *n* = 3.

The lowest mobility was determined in 2% MMW chitosan gel (−0.39 ± 0.22 g*s), slightly higher in 2% HMW 80/1000 chitosan gel (−0.83 ± 0.09 g*s), but there was no significant difference between these two gels. The maximum mobility had 2% HMW 80/3000 chitosan gel (2.99 ± 0.74 g*s). The results of this gel are significantly higher than those of the previous samples. Comparing MMW chitosan gels of different concentrations, it was found that 4% gel had a significantly higher mobility (4.78 ± 0.1 g*s) than 2% MMW gel. Figure 7 shows adhesion (g*s) results of the gels.

When evaluating the adhesion (g*s) of chitosan gels with different molecular weights at the same concentration, the 2% MMW chitosan gel had the lowest adhesion (−1.76 ± 0.04 g*s), 2% HMW 80/1000 chitosan gel had higher adhesion (−2.57 ± 0.23 g*s), and 2% HMW 80/3000 chitosan gel had the highest adhesion (−4.68 ± 0.68 g*s). The differences between these results are significant. The 4% MMW chitosan gel had significantly higher adhesion (−7.69 ± 0.19 g*s) compared to 2% MMW chitosan gel. Another study also prepared gels containing chitosan with different molecular weights (low molecular weight (LMW), MMW, and HMW). After analysis of their texture, the parameters of hardness, consistency, and stickiness were evaluated. MMW chitosan gel was found to have the lowest values for all of the listed properties, HMW chitosan gel the highest, and LMW chitosan gel fell in between [38]. Comparing these properties to the MMW and HMW gels prepared in our study, the obtained results are similar. The scientific literature also contains a study in which chitosan gel hardness and stickiness increased with increasing chitosan concentrations [39]. According to the results of Sezer et al. [36], the molecular weight and concentration of the chitosan used in the gel can influence the adhesion of the formulation.

Summarizing the results for hardness, consistency, stickiness, viscosity index, mobility, and adhesion of chitosan gels of different molecular weights, it can be concluded that 2% MMW chitosan gel had the lowest texture properties, 2% HMW 80/1000 chitosan gel had higher texture properties, and the 2% HMW 80/3000 chitosan gel had the highest. Evaluating the results for chitosan gels with different concentrations, it was found that a higher concentration of chitosan produced higher values for these texture parameters.

### 3.3. Formation of Microcapsules

**Figure 8:**
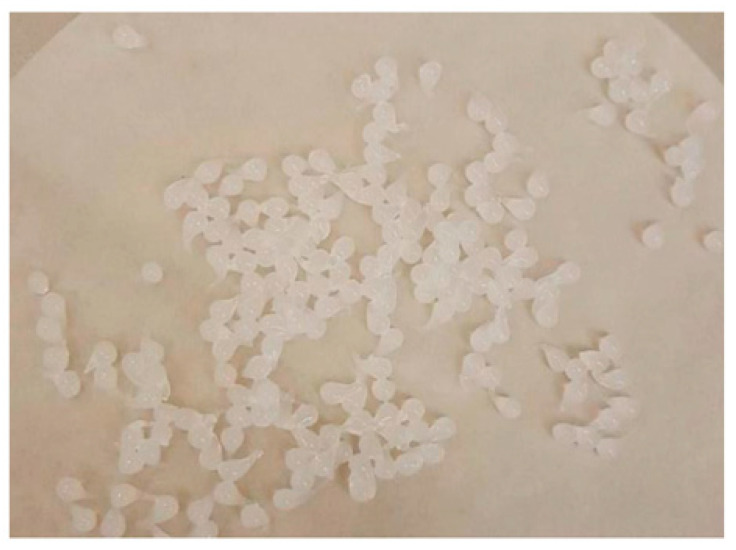
2% HMW 80/1000 chitosan gel microcapsules formed when dropped from a distance of 4 cm.

**Figure 9:**
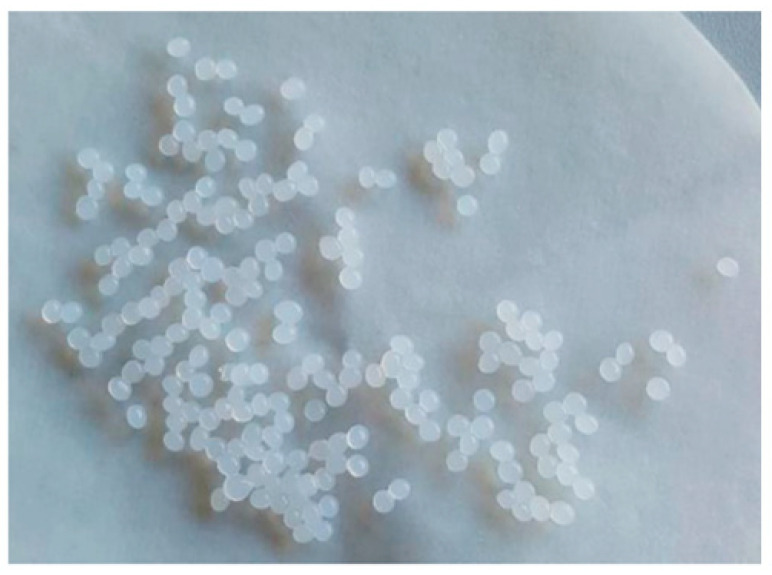
2% HMW 80/1000 chitosan gel microcapsules formed when dropped from a distance of 10 cm.

The ability to form a gel in contact with anionic groups is an interesting feature of chitosan. This gel formation process is due to the presence of anionic groups that allow the formation of intrachain and interchain cross-links [40]. Different technological factors were assessed to determine the optimal conditions for the formation of microcapsules. Initially, we chose to form microcapsules from 2% MMW and 2% HMW 80/1000 chitosan gels using different gel release heights (4 and 10 cm) into a 1% NaOH solution with different mixing times (5, 15, and 30 min). It was found that more regular microcapsules were formed by releasing the gel from a 10 cm height. When the gel was dripped from 4 cm, the microcapsules formed an elongated shape instead of a sphere. Figure 8 and Figure 9 show microcapsules formed from 2% HMW 80/1000 chitosan gel released from 4 and 10 cm height into a NaOH solution.

Chitosan gels with 4% MMW and 2% HMW 80/3000 did not form microcapsules. The main reason for this was the viscosity of these gels was too high to form droplets. Thus we produced microcapsules from a 2% MMW chitosan gel using a syringe pump with a dripping speeds and different stirring times.

Microcapsules were formed from emulsions consisting of 2% MMW chitosan gel, 0.5% Tween 20, and different concentrations of *Boswellia serrata* essential oil. Emulsions that dripped at 0.8 mL/min speed formed round-spherical (regular) shape microcapsules with 0.1, 0.2, and 0.3% essential oil, whereas irregular microcapsules formed with 0.4% essential oil. By reducing the dripping speed of the emulsion to 0.4 mL/min, regular microcapsules were formed with 0.4% essential oil, but the microcapsules did not form properly with higher oil concentrations. Microcapsules were found to be more regular when the emulsion was dripped at 0.4 mL/min speed, which was selected for further studies. A 15 min stirring time was chosen to produce microcapsules in the following experiments.

We tested different emulsifiers in the emulsion composition. Emulsions consisting of 2% MMW chitosan gel, 0.5% Tween 80 and different concentrations of *Boswellia serrata* essential oil only produced suitable microcapsules with 0.1% of the essential oil. Span 20 and Span 80 emulsifiers could be used to encapsulate 0.1% and 0.2% of the essential oil, and when MMW was replaced by HMW 80/1000 chitosan, Tween 20 was selected to encapsulate *Boswellia serrata* essential oil at a concentration from 0.1% to 0.4%. Microcapsules could not be formed from an emulsion containing 3% MMW chitosan, 0.1% *Boswellia serrata* essential oil, and 0.5% Tween 20 because the emulsion was too viscous.

The results show that the most regular microcapsules were formed from 2% chitosan emulsion containing MMW or HMW 80/1000 when the optimal drip rate was 0.4 mL/min and the concentration of *Boswellia serrata* essential oil was 0.4%.

### 3.4. Influence of Different Technological Factors on the Appearance and Size of Microcapsules

**Figure 10:**
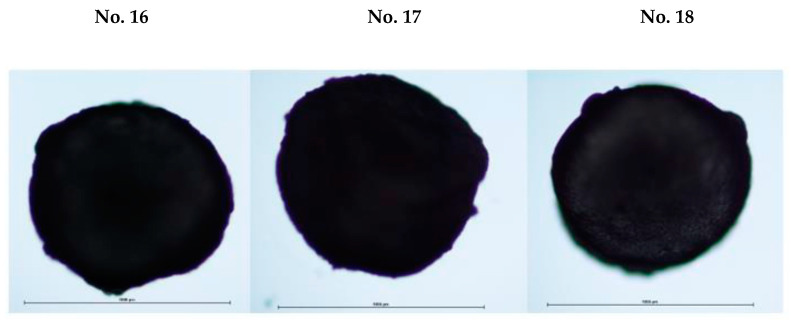
Microcapsule **No. 16**, **No. 17**, and **No. 18** microscopic images magnified 100 times (size about 100 µm). The composition of the microcapsules according to their number are presented in Table 3.

After drying the prepared microcapsules, the influence of different technological factors (concentration of *Boswellia serrata* essential oil, mixing time, emulsifiers, different molecular weight of chitosan) on the appearance and size of microcapsules was evaluated. The shape and appearance of microcapsules can affect the following properties: mechanical strength, degree of swelling, and the protection and release of the encapsulated bioactive compounds [41]. Microscopic examination of the appearance of dried microcapsules revealed that they all have a slightly rough surface, with slight irregularities, in the shape of an irregular circle. A microscopic image of microcapsules No. 16, No. 17, and No.18 is shown in Figure 10.

In order to compare the size of the produced and dried microcapsules and its dependence on *Boswellia serrata* essential oil concentration, we used a single chitosan gel composition, emulsifier, and microcapsule mixing time. The sizes of microcapsules from different batches are given in Table 5.

When comparing microcapsule diameters, no significant difference was found, except for microcapsules No. 6 and No. 12 (*p* < 0.05). Microcapsule No. 6 and No. 12 contained 0.1% and 0.3% of *Boswellia serrata* essential oil, respectively. In this example, microcapsules containing less essential oil were found to have a larger diameter (974.09 ± 26.78 μm) than microcapsules containing a higher concentration of essential oil (943.82 ± 31.47 μm).

To evaluate the effect of stirring time on the diameter of the microcapsules, microcapsules with the same composition and technological factors except stirring time were compared. Microcapsules formed at three different stirring times (5, 15, and 30 min) were evaluated. This technological factor was found to cause significant differences in microcapsule diameters. The diameter of the microcapsules decreased as the stirring time increased. For example, microcapsule No. I (stirring time, 5 min) had a diameter of 1087.39 ± 53.43 μm compared to 1002.51 ± 8.76 μm for microcapsule No. II (stirring time, 15 min) and 973.42 ± 15.73 μm for microcapsule No. III (stirring time, 30 min).

To evaluate the influence of emulsifiers on the diameter of microcapsules, microcapsules were formed using different emulsifiers. The largest significant difference in the diameter was found to be between the microcapsules containing 0.5% Tween 20 and the other emulsifiers. Microcapsule No. 19, which contained 0.5% Tween 80, was significantly smaller than microcapsule No. 5 (containing 0.5% Tween 20). Compared to microcapsules containing other emulsifiers, microcapsule No. 19 was significantly larger than microcapsule No. 22 (containing 0.5% Span 80) but not microcapsule No. 20 (containing 0.5% Span 20). The smallest of all microcapsules, No. 22 and No. 23, contained Span 80 and had a diameter of 846.98 ± 8.38 μm and 850.48 ± 3.41 μm, respectively.

No significant difference was found between the batches of microcapsules consisting of MMW or HMW 80/1000 chitosan. For example, microcapsule No. 1, containing 2% MMW chitosan, was 1091.09 ± 9.12 μm in size, and microcapsule No. 25, containing 2% HMW 80/1000 chitosan, was 1010.66 ± 3.84 μm. Dima et al. [6] produced microcapsules that were 1224 ± 6.56 μm in a diameter. The larger microcapsule size may have been due to slightly different study conditions, e.g., their microcapsules contained not only chitosan but also the polysaccharide carrageenan, and Tween 40 was used as an emulsifier. In addition, their microcapsules were stirred in ethanol instead of aqueous NaOH, and ethanol has been shown to reduce the solubility and increase the hardness of chitosan [6]. Hu et al. [23] analyzed microcapsules formed from a lower concentration of chitosan (0.5%). The microcapsules were stirred at 400, 800, and 1500 rpm, and their sizes were 225 ± 4 μm, 131 ± 20 μm, and 11 ± 3 μm, respectively. Thus, higher stirring speeds produces smaller microcapsules [42]. In our study, the stirring speed was fixed at 130 rpm. It has been determined that smaller size microcapsules may increase the release of essential oil components [43]. Emulsifiers in the emulsion system form a protective membrane between the aqueous and oil phases, thus making the system more stable. Yang et al. [25] found that a complex of two emulsifiers (Span 80 and Tween 60) enhanced the membrane by increasing the viscosity of the interface. This might account for a decrease in microcapsule diameter from 626.5 μm (using Span 80) to 31.8 μm (using Span 80 and Tween 60) [44].

Javid et al. [26] produced microcapsules containing chitosan and eucalyptus or sandalwood essential oils. It was found that as the concentration of eucalyptus essential oil increased, the size of the microcapsules also increased, but the size decreased when using higher concentrations of sandalwood essential oil [45]. In our study, different concentrations of *Boswellia serrata* essential oil did not significantly change microcapsule size.

Summarizing our results, we can state that significant changes in the size of microcapsules was not caused by a change in the concentration of *Boswellia serrata* essential oil or the molecular weight of chitosan. Emulsifiers did have an effect on the size of the microcapsules, where the largest microcapsules were formed with Tween 20, and the smallest ones with Span 80. The microcapsules containing Span 20 and Tween 80 were arranged in a descending order, respectively.

### 3.5. Influence of Boswellia serrata Essential Oil Concentration on the Hardness of Microcapsules

Mechanical compression is a good way to determine the quality of produced microcapsules [46]. The mechanical properties of microcapsules are highly dependent on the physical properties of their shell. Polymeric materials are often used to form microcapsules to increase their compressive strength [47]. To evaluate the mechanical properties of microcapsules, the methods can test a single microcapsule or a group of microcapsules. Examination of a single microcapsule gives more accurate results [48]. The literature also states that the compressive force on microcapsules between two parallel plates cannot predict their mechanical properties in another medium, such as when the microcapsules are suspended in a flowing liquid. The motion and deformation of the particles then depends not only on their physical properties (internal rheology, surface-volume ratio, mechanical properties of the shell) but also on the local field flow [49]. In our study, it was not possible to evaluate just one microcapsule in a compression test, so a group of 10 microcapsules from each batch was tested by repeating the test 3 times.

**Figure 11:**
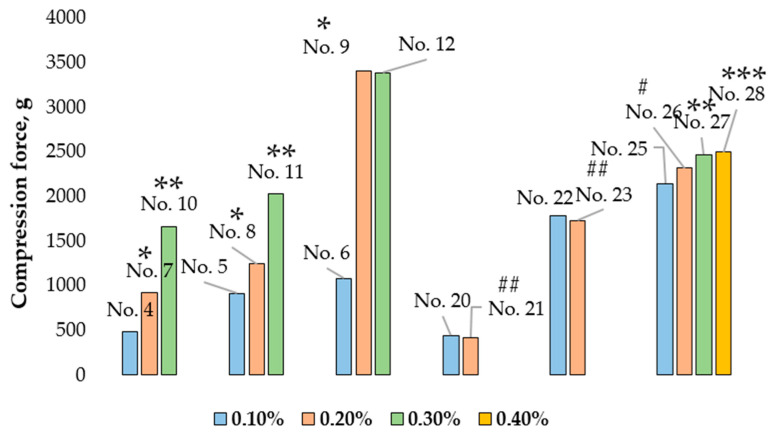
Influence of *Boswellia serrata* essential oil concentration on microcapsule hardness. * *p* < 0.05 vs. microcapsules containing 0.1% essential oil, ** *p* < 0.05 vs. microcapsules containing 0.2% essential oil, *** *p* > 0.05 vs. microcapsules containing 0.3% essential oil, # *p* > 0.05 vs. No. 9, ## *p* > 0.05 vs. microcapsules containing 0.1% essential oil; *n* = 3. The composition of the microcapsules according to their number is presented in Table 3.

Compression of microcapsules containing 0.1% of the essential oil (No. 4) required 483.54 ± 89.79 g compression force; microcapsules containing 0.2% of the essential oil (No. 7) required 922.26 ± 254.65 g; and those containing 0.3% of the essential oil (No. 10) required 1665.73 ± 439.40 g (Figure 11). These microcapsules contained 2% MMW chitosan and 0.5% Tween 20, the stirring time was 5 min, and the differences were significant. Microcapsules No. 5, No. 8, and No. 11 were also examined in the shell compression test. These microcapsules contained 2% MMW chitosan and 0.5% Tween 20, but the stirring time was 15 min. A significant difference was found between the results when comparing essential oil content. The compression force required to crush No. 5 (containing 0.1% essential oil) was 913.22 ± 90.87 g compared to 1242.78 ± 249.45 g for No. 8 (containing 0.2% of essential oil) and 2034.83 ± 208.59 g for No. 11 (containing 0.3% of essential oil). In the evaluation of microcapsules No. 6 (0.1% essential oil), No. 9 (0.2% essential oil), and No. 12 (0.3% essential oil), No. 6 (1078.87 ± 152.41 g) was the least resistant to compression followed by No. 12 (3385.58 ± 266.66 g), whereas the highest compression force was required for No. 9 (3404.58 ± 577.39 g). All these microcapsules contained 2% MMW chitosan and 0.5% Tween 20, and the mixing time was 30 min. No significant difference was found when comparing microcapsules No. 20 (0.1% essential oil) and No. 21 (0.2% essential oil), which contained 2% MMW chitosan and 0.5% Span 20, or No. 22 (0.1% essential oil) and No. 23 (0.2% essential oil), which contained 2% MMW chitosan and 0.5% Span 80. Higher concentrations of *Boswellia serrata* essential oil positively influenced the resistance of microcapsules No. 25, No. 26, No. 27, and No. 28, which contained 2% HMW 80/1000 chitosan and 0.5% Tween 20.

Summarizing the results, it can be concluded that increasing concentrations of *Boswellia serrata* essential oil significantly increased the resistance of the microcapsule shell to crushing when the microcapsules contained 2% MMW chitosan and 0.5% Tween 20.

### 3.6. Influence of Stirring Time on the Hardness of Microcapsules

**Figure 12:**
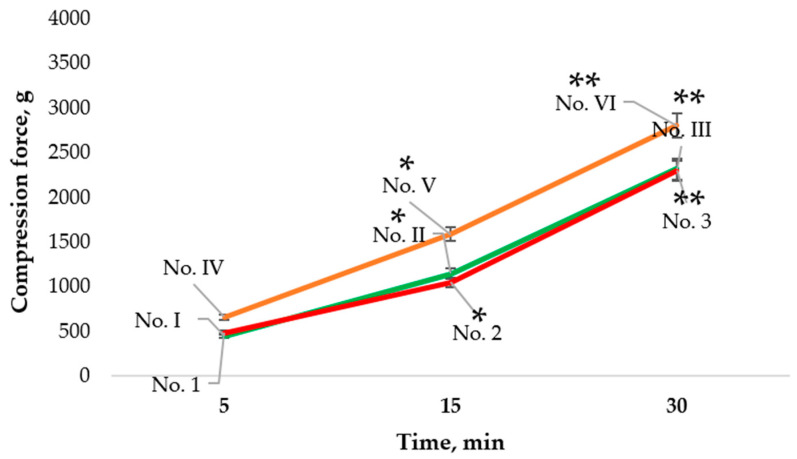
Influence of stirring time on the hardness of microcapsules formed from chitosan gels. * *p* < 0.05 vs. microcapsules stirred for 5 min, ** *p* < 0.05 vs. microcapsules stirred for 15 min; *n* = 3. The composition of the microcapsules according to their number is presented in Table 1, Table 2 and Table 3.

Figure 12 shows that the hardness of the microcapsules containing 2% MMW or 2% HMW 80/1000 chitosan gel changed depending on the stirring time. The green curve represents the results for microcapsules No. I (stirring time 5 min), No. II (stirring time 15 min), and No. III (stirring time 30 min). These microcapsules were formed from 2% MMW chitosan gel without the aid of a syringe pump.

The compression force needed to crush the shell of microcapsule No. I was 449.24 ± 27.44 g compared to 1143.43 ± 100.43 g for No. II and 2315.80 ± 103.48 g for No. III. The difference between the results was significant. A significant difference was also found between the hardness of microcapsules No. IV (stirring time 5 min), No. V (stirring time 15 min), and No. VI (stirring time 30 min) (orange curve in Figure 12 which were prepared from 2% HMW 80/1000 chitosan gel without a syringe pump. Once again, as the stirring time for the microcapsules lengthened, their resistance to crushing increased. Microcapsules No. 1 (stirring time 5 min), No. 2 (stirring time 15 min), and No. 3 (stirring time 30 min) (red curve in Figure 12) were prepared from 2% MMW chitosan gel using a syringe pump and the obtained data were very similar to that for microcapsules No. I, No. II, and No. III.

**Figure 13:**
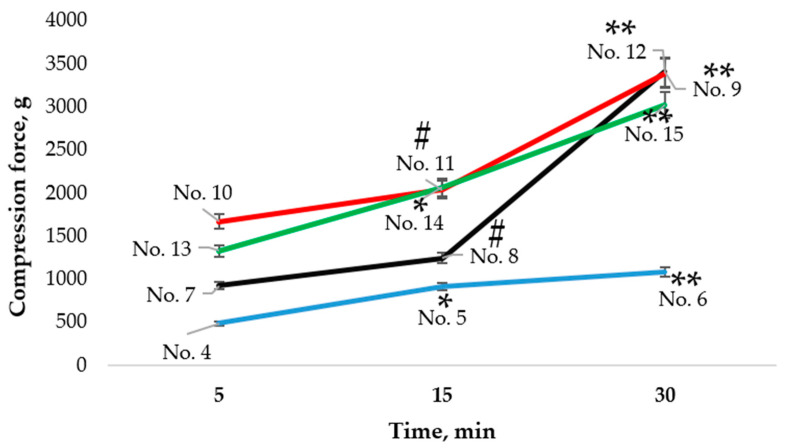
Influence of stirring time on the hardness of microcapsules formed from emulsions. * *p* < 0.05 vs. microcapsules stirred for 5 min, ** *p* < 0.05 vs. microcapsules stirred for 15 min, # *p* > 0.05 vs. microcapsules stirred for 5 min; *n* = 3. The composition of the microcapsules according to their number is presented in Table 3.

The hardness results for microcapsules formed from emulsions containing 0.1% *Boswellia serrata* essential oil, 2% MMW chitosan, 0.5% Tween 20 that were prepared with different stirring times are shown in Figure 13. Comparing the microcapsules No. 4 (stirring time 5 min), No. 5 (stirring time 15 min), and No. 6 (stirring time 30 min), it was found that as the stirring time lengthened, the microcapsules required more force to crush them, though the difference between No. 5 and No. 6 was not statistically significant.

Microcapsules No. 7 (stirring time 5 min), No. 8 (stirring time 15 min), and No. 9 (stirring time 30 min), which contained 0.2% *Boswellia serrata* essential oil, 2% MMW chitosan, and 0.5% Tween 20, also needed a higher force to crush them as the stirring time increased. Microcapsules No. 9 were harder than No. 7 and No. 8 (*p* < 0.05).

Microcapsules No. 10 (stirring time 5 min), No. 11 (stirring time 15 min), and No. 12 (stirring time 30 min) were formed from 0.3% *Boswellia serrata* essential oil, 2% MMW chitosan, and 0.5% Tween 20. The softest microcapsules in this group was No. 10, and No. 12 was the hardest.

Microcapsules No. 13 (stirring time 5 min), No. 14 (stirring time 15 min), and No. 15 (stirring time 30 min) contained 0.4% *Boswellia serrata* essential oil, 2% MMW chitosan, and 0.5% Tween 20. As the stirring time increased, the microcapsules became increasingly harder to crush.

Summarizing the obtained results, we can state that microcapsules that consisted only of chitosan gel were stronger when stirred for longer time. Microcapsules composed of emulsions did not always show significant differences in hardness with longer stirring times, but there was a tendency for it to increase as the stirring time increased.

### 3.7. Influence of Surfactants on the Hardness of Microcapsules

**Figure 14:**
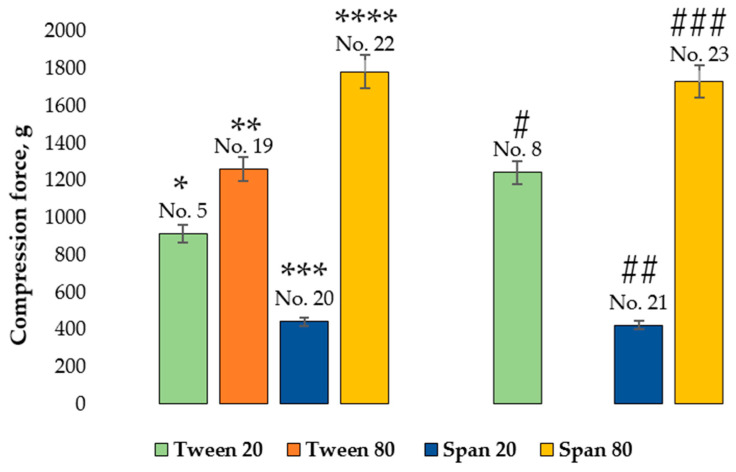
Influence of emulsifiers on hardness of microcapsules. * *p* > 0.05 vs. No. 19, ** *p* < 0.05 vs. No. 20, *** *p* < 0.05 vs. No. 5, **** *p* < 0.05 vs. No. 5, No. 19, No. 20, # *p* > 0.05 vs. No. 23, ## *p* < 0.05 vs. No. 8, ### *p* < 0.05 vs. No. 21; *n* = 3. The composition of the microcapsules according to their number is presented in Table 3.

In our study, microcapsules were formed with four different emulsifiers: Tween 20, Tween 80, Span 20, and Span 80. The results for their effect on hardness are shown in Figure 14.

Comparing microcapsules No. 5, No. 19, No. 20, and No. 22 by the shell hardness, the lowest compressive forces were required to crush microcapsules containing Span 20 (microcapsules No. 20). A statistically significant difference in the hardness of microcapsules was found between No.20 and No. 5, No. 19, and No. 22 samples (*p* < 0.05). The highest value of hardness was observed for microcapsules No. 5, containing 0.5% Tween 20 (913.22 ± 90.87 g), followed by No. 19 with Tween 80 (1262.26 ± 397.43 g), and No. 22 with Span 80 (1782.50 ± 863.91 g). There were no significant differences between the results for these microcapsules. All microcapsules contained 0.1% *Boswellia serrata* essential oil and 2% MMW chitosan.

Because only one batch of microcapsules containing 0.1% *Boswellia serrata* essential oil was formed with Tween 80, for further comparison we chose microcapsules No. 8 (Tween 20), No. 21 (Span 20), and No. 23 (Span 80). All of these microcapsules contained 0.1% *Boswellia serrata* essential oil and 2% MMW chitosan. As in previous group of comparisons, the softest group of microcapsules was No. 21 with Span 20 (123.63 ± 12.67 g) and the hardest was No. 23 with Span 80 (1729.39 ± 240.08 g).

Summarizing the results, we can state that the softest microcapsules are formed containing Span 20. Microcapsules with a stronger coating can be formed using Tween 20, Tween 80 or Span 80, and the differences between the hardness with these emulsifiers are insignificant.

### 3.8. Influence of Different Molecular Weights of Chitosan on the Hardness of Microcapsules

**Figure 15:**
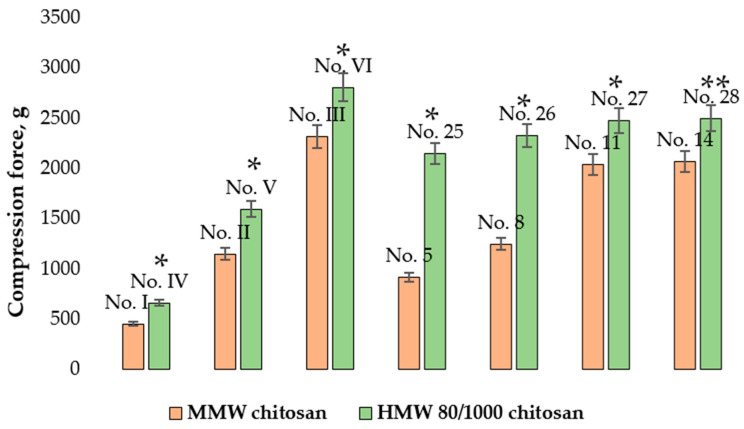
Influence of chitosan molecular weight on microcapsule hardness. * *p* < 0.05 vs. microcapsules containing MMW chitosan, ** *p* > 0.05 vs. microcapsules containing MMW chitosan; *n* = 3. The composition of the microcapsules according to their number is presented in Table 1, Table 2 and Table 3.

Figure 15 shows the crushing results for chitosan microcapsules prepared from different molecular weight chitosan gels.

In all cases, microcapsules containing HMW 80/1000 chitosan were harder than microcapsules containing MMW chitosan. This was due to the higher value of HMW 80/1000 gel hardness determined in the texture study.

### 3.9. Moisture Content in Microcapsules

**Figure 16:**
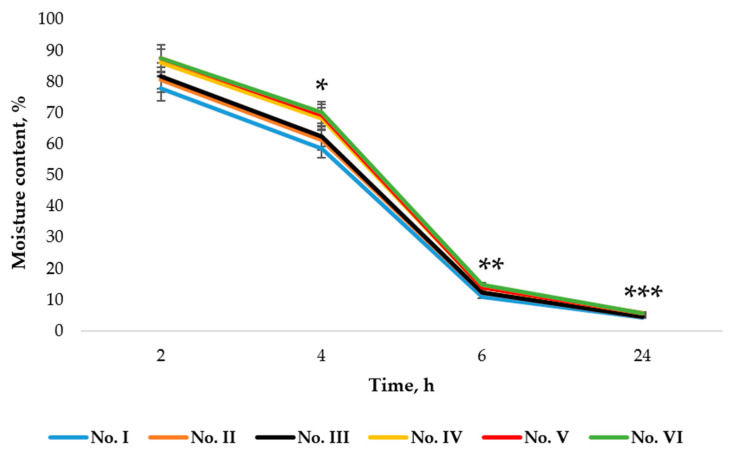
Dependence of microcapsule moisture content on drying time. * *p* < 0.05 vs. moisture content after 4 h, ** *p* < 0.05 vs. moisture content after 6 h, *** *p* < 0.05 vs. moisture content after 24 h; *n* = 3. The composition of the microcapsules according to their number is presented in Table 1.

Moisture content is an important factor when assessing the viability of microorganisms in a product [50]. The results are shown in Figure 16.

Freshly made microcapsules No. I, No. II, and No. III (containing MMW chitosan) and microcapsules No. IV, No. V, and No. VI (containing HMW 80/1000 chitosan) were compared at 2, 4, 6, and 24 h after production. A significant difference was observed in the moisture content of the microcapsules measured at different times. The moisture content of the microcapsules decreased with time. After 2 h the moisture content varied from 77.82% ± 0.99 to 87.54% ± 0.51; by 4 h, the moisture content ranged from 58.44% ± 0.72 to 70.10% ± 0.63; by 6 h, it was found to be 11.04% ± 0.34 to 14.80% ± 0.22; and after 24 h, the moisture content ranged from 4.27% ± 0.28 to 5.6% 2 ± 0.38. This means that the microcapsules lost the largest amount of moisture during the first 6 h of drying, whereas the loss over the next 18 h was much smaller. A significant difference was also found between the moisture content of microcapsules prepared by using chitosan gels with different molecular weights. Microcapsules containing HMW 80/1000 chitosan had a higher moisture content than microcapsules containing MMW chitosan. The moisture content at 24 h in microcapsules with MMW chitosan ranged from 4.27% ± 0.28% to 4.47% ± 0.19 compared to 5.65% ± 0.33 and 5.68% ± 0.04 for HMW 80/1000 chitosan.

The results for moisture content measured 24 h after microcapsule formation are shown in Table 6.

The largest difference in the moisture content of the dried microcapsules was observed for different molecular weights of chitosan (*p* < 0.05). In microcapsules containing MMW chitosan, the moisture content ranged from 4.21% ± 0.36% to 4.66% ± 0.39, and in microcapsules containing HMW 80/1000 chitosan, the moisture content ranged from 5.44% ± 0.36 to 5.69% ± 0.45.

In a previous study, microcapsules with several compositions were produced by a spraying method. The moisture content of dried microcapsules containing 4% chitosan was 2.17% ± 0.11 [41]. The lower moisture content in these microcapsules compared to our results may have been due to the higher concentration of chitosan and a different production method. In the same study, microcapsules containing inulin had a higher moisture content of 4.38% ± 0.31. Inulin has been shown to absorb higher amounts of water [41].

Summarizing the results, we can say that microcapsules lost the largest amount of moisture between 4 and 6 h after preparation. After the final drying, the moisture content of microcapsules with MMW chitosan varied from 4.21% ± 0.36% to 4.66% ± 0.39 and that of microcapsules with HMW 80/1000 chitosan varied from 5.44% ± 0.36 to 5.69% ± 0.45, and this difference was statistically significant.

### 3.10. Composition of Boswellia serrata Essential Oil

A total of 50 compounds was identified in *Boswellia serrata* essential oil by GC-MS. The main components were α-pinene (46.90%), α-felandren (25.41%), tricyclo [3.1.1.03.6] heptane-6-carboxylic acid (6.00%), sabinene (4.17%), limonene (2.73%), and anisole (2.16%) (Supplementary Table S1). Gupta et al. [51] prepared *Boswellia serrata* essential oil from *Boswellia* raw material collected from three different locations for analysis by GC-MS. The highest content of active compounds in these essential oils was α-thujene (22.5–69.8%) and α-pinene (3.5–10.9%). They also found sabinene (2.5–5.9%) and limonene (1.3–3.7%), which were predominant in the essential oil composition. Ayub et al. [26] identified 21 compounds in *Boswellia serrata* essential oil, including α-pinene (77.95–89.07%), α-thujene (3.72–4.49%), trans-verbenol (0.68–2.48%), β-thujone (0.86–2.21%), *p*-cymene (2.11–0.93%), *m*-cymene (0.89–1.89 %), and sabinene (0.93–1.66%). Iram et al. [52] summarized that the essential oil of *Boswellia serrata* mainly contains monoterpenoids (α-pinene, trans-verbenol, trans-pinocarveol, borneol, myrcene, phallendrene, cadinene, verbenone, limonene, thuja2,4(10)-diene and *p*-cymene) and small amount of diterpenes. The composition of an essential oil highly depends on the geographical location from which the raw material is obtained [51,53].

### 3.11. Emulsion Microscopy and Evaluation of Stability

**Figure 17:**
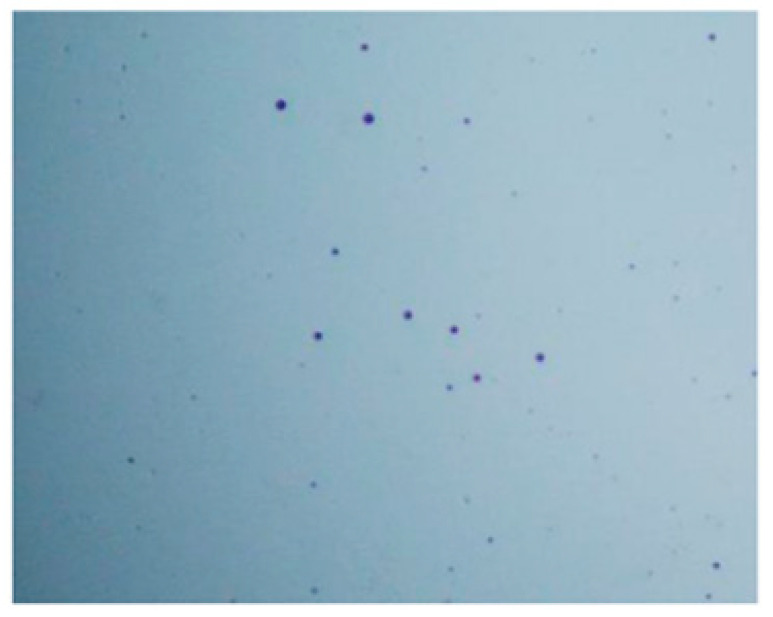
Emulsion No. 1 microscopic image magnified 100 times.

**Figure 18:**
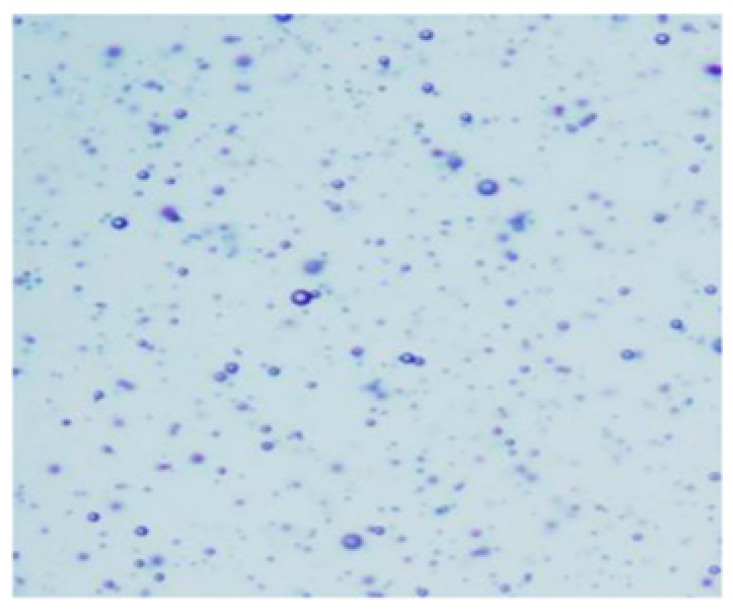
Emulsion No. 4 microscopic image magnified 100 times.

After microscopic examination of the emulsion samples, the droplet sizes of Boswellia serrata essential oil were measured and their distribution in the emulsion was visually compared. The droplets were more densely distributed in emulsions with a higher concentration of the essential oil. A microscopic view of emulsion No. 1 (containing 2% MMW chitosan, 0.1% essential oil, and 0.5% Tween 20) is shown in Figure 17 and emulsion No. 4 (containing 2% MMW chitosan, 0.4% essential oil, and 0.5% Tween 20) is shown in Figure 18.

The droplet size of the emulsion is an important factor influencing the stability of the emulsion and its texture [54]. Measurement of the droplet size in our emulsions showed that the mean droplet size increased as the concentration of the essential oil increased, but there was no significant difference between the results. Another study has shown that droplet size also increases as the concentration of essential oil increases in the emulsions [55]. In our study, no significant difference was found between the results when comparing emulsions prepared with different emulsifiers. The results are shown in Table 7.

The stability of the emulsions was tested with centrifugation, which showed that all emulsions were stable. The oil-phase and aqueous phase did not separate when the emulsions were centrifuged for 5 min at 10,000 rpm. The emulsions were stable because the chitosan molecules were adsorbed at the oil-water interface, which improved emulsion stability [54].

To summarize the results, as concentration of the essential oil in the emulsions increased, the droplets were more densely distributed, and their size tended to increase without any significant differences. All of the examined emulsions were stable.

## 4. Conclusions

Our texture analysis of chitosan gels revealed that different molecular weights and concentrations of chitosan affect the values of gel hardness, consistency, stickiness, viscosity index, mobility, and adhesion. Compared to MMW and HMW chitosan gels, HMW 80/3000 chitosan gel showed significantly higher results for the texture properties. It was also found that increasing the concentration of chitosan from 2 to 4% significantly increased gel hardness, consistency, stickiness, viscosity index, mobility, and adhesion. It was found that microcapsules with a regular shape were formed with either Tween 20 and 0.1 to 0.4% *Boswellia serrata* essential oil, Tween 80 and up to 0.1% *Boswellia serrata* essential oil, or Span 20 and Span 80 and 0.1 to 0.2% *Boswellia serrata* essential oil. Spherical microcapsules were formed using MMW and HMW 80/1000 chitosan. Microcapsules with HMW 80/1000 chitosan were harder and contained more moisture than microcapsules with MMW chitosan (*p* < 0.05). When the stirring time for the microcapsules was increased to 30 min, their diameter decreased significantly and they retained a regular oval shape. The resistance of microcapsule shell to crushing increased significantly with the amount of stirring. The compression test also showed that increasing concentrations of essential oil increased the resistance of microcapsules containing 2% MMW chitosan and 0.5% Tween 20 (*p* < 0.05). Our study results will be important for other researchers that are working with natural active substances and microencapsulation methods. For future scope, it will be necessary to analyze the release of active ingredients (for example, *Boswellia serrata* essential oil), and perform a thermogravimetric analysis to obtain the best heat resistance with a constant rate of weight loss and the highest encapsulation efficiency for the potential control of the releasing property during processing. In addition, there is huge interest from industry companies that want to improve their products with active substances and apply new technologies to their manufacturing processes. Coacervation is an easily applicable encapsulation method for the food, cosmetic, and pharmaceutical industries. New active compounds, products, and innovative and simple technologies will help manufacturers and distributors become leaders in the global market.

## Figures and Tables

**Table 1:** Composition of microcapsules and technological factors of their production.

Microcapsule	Chitosan Concentration, %	Stirring Time, min	Launch Height, cm
I	2%; MMW	5	10
II	2%; MMW	15	10
III	2%; MMW	30	10
IV	2%; 80/1000 HMW	5	10
V	2%; 80/1000 HMW	15	10
VI	2%; 80/1000 HMW	30	10
VII	2%; HMW	5	4
VIII	2%; HMW	15	4
IX	2%; HMW	30	4
X	4%; HMW	5	4
XI	2%; 80/1000 MMW	5	4
XII	2%; 80/1000 MMW	15	4
XIII	2%; 80/1000 MMW	30	4
XIV	2%; 80/3000 MMW	5	4

**Table 2:** Composition of emulsions.

No. of Emulsion	Concentration of Chitosan Gel, %	Concentration of *Boswellia serrata* Essential Oil, %	Concentration of Emulsifiers, %
1	2% MMW	0.1%	Tween 20; 0.5%
2	2% MMW	0.2%	Tween 20; 0.5%
3	2% MMW	0.3%	Tween 20; 0.5%
4	2% MMW	0.4%	Tween 20; 0.5%
5	2% MMW	0.1%	Tween 80; 0.5%
6	2% MMW	0.1%	Span 20; 0.5%
7	2% MMW	0.2%	Span 20; 0.5%
8	2% MMW	0.1%	Span 80; 0.5%
9	2% MMW	0.2%	Span 80; 0.5%
10	3% MMW	0.1%	Tween 20; 0.5%
11	2% HMW 80/1000	0.1%	Tween 20; 0.5%
12	2% HMW 80/1000	0.2%	Tween 20; 0.5%
13	2% HMW 80/1000	0.3%	Tween 20; 0.5%
14	2% HMW 80/1000	0.4%	Tween 20; 0.5%
15	2% HMW 80/1000	0.5%	Tween 20; 0.5%

**Table 3:** Formulations of microcapsules formed by a syringe pump.

Microcapsule	Chitosan Gel	No. of Emulsion *	Stirring Time, min	Drip Rate, mL/min
1	2% MMW	-	5	0.5
2	2% MMW	-	15	0.5
3	2% MMW	-	30	0.5
4	-	1	5	0.8
5	-	1	15	0.8
6	-	1	30	0.8
7	-	2	5	0.8
8	-	2	15	0.8
9	-	2	30	0.8
10	-	3	5	0.8
11	-	3	15	0.8
12	-	3	30	0.8
13	-	4	5	0.8
14	-	4	15	0.8
15	-	4	30	0.8
16	-	4	5	0.4
17	-	4	15	0.4
18	-	4	30	0.4
19	-	5	15	0.4
20	-	6	15	0.4
21	-	7	15	0.4
22	-	8	15	0.4
23	-	9	15	0.4
24	-	10	15	0.4
25	-	11	15	0.4
26	-	12	15	0.4
27	-	13	15	0.4
28	-	14	15	0.4

* Emulsion numbers correspond to those presented in Table 2.

**Table 4:** pH Values of chitosan gels.

Gel	pH Value
2% MMW chitosan gel	3.37 ± 0.03
4% MMW chitosan gel	3.65 ± 0.03 *
2% HMW 80/1000 chitosan gel	3.34 ± 0.05 **
2% HMW 80/3000 chitosan gel	3.35 ± 0.04 ***

* *p* < 0.05 vs. 2% MMW chitosan gel, ** *p* > 0.05 vs. 2% MMW chitosan gel, *** *p* > 0.05 vs. 2% HMW 80/1000 chitosan gel; *n* = 3.

**Table 5:** Size of microcapsules.

No. of Microcapsule	Microcapsule Size, μm
I	1087.39 ± 53.43
II	1002.51 ± 8.76 *
III	973.42 ± 15.73 **
IV	1090.45 ± 41.27
V	1024.68 ± 19.85 *
VI	938.61 ± 36.85 **
1	1091.09 ± 9.12
2	1009.32 ± 14.76 *
3	964.91 ± 8.68 **
4	1111.39 ± 13.57
5	1011.23 ± 12.61 *
6	974.09 ± 26.78 **
7	1116.25 ± 7.92 ***
8	1017.35 ± 16.69 * ***
9	976.32 ± 9.58 ** ***
10	1090.31 ± 38.33 ****
11	1009.39 ± 6.95 * ****
12	943.82 ± 31.47 ** **** #
16	1098.12 ± 24.80 *****
17	1012.35 ± 11.87 * *****
18	955.94 ± 12.04 ** *****
19	941.98 ± 28.16 ## ###
20	878.52 ± 19.03
21	874.12 ± 4.56 *
22	846.98 ± 8.38 ####
23	850.48 ± 3.41 * #####
25	1010.66 ± 3.84 †
26	1013.52 ± 10.68 * †
27	1014.81 ± 11.94 ** †
28	1016.88 ± 10.23 *** †
17	1012.35 ± 11.87 * *****
18	955.94 ± 12.04 ** *****

* *p* < 0.05 vs. microcapsules mixed for 5 min, ** *p* < 0.05 vs. microcapsules mixed for 15 min, *** *p* > 0.05 vs. microcapsules containing 0.1% essential oil, **** *p* > 0.05 vs. microcapsules containing 0.2% essential oils, ***** *p* > 0.05 vs. microcapsules containing 0.3% essential oil, # *p* < 0.05 vs. No. 6, ## *p* < 0.05 vs. No. 5 and No. 22, ### *p* > 0.05 vs. No. 20, #### *p* < 0.05 vs. No. 20, ##### *p* < 0.05 vs. No. 21, † *p* > 0.05 vs. microcapsules containing MMW chitosan; *n* = 3. The composition of the microcapsules according to their number is presented in Table 3.

**Table 6:** Moisture content of microcapsules after 24 h drying.

No. of Microcapsule	Moisture Content 24 h after Production, %
1	4.29 ± 0.65
2	4.21 ± 0.36
3	4.36 ± 0.25
4	4.51 ± 0.54
5	4.36 ± 0.26
6	4.28 ± 0.65
7	4.27 ± 0.45
8	4.66 ± 0.39
9	4.23 ± 0.22
10	4.37 ± 0.29
11	4.31 ± 0.57
12	4.59 ± 0.36
16	4.27 ± 0.59
17	4.56 ± 0.64
18	4.45 ± 0.12
19	4.26 ± 0.65
20	4.62 ± 0.31
21	4.56 ± 0.36
22	4.35 ± 0.45
23	4.65 ± 0.24
25	5.69 ± 0.45 *
26	5.44 ± 0.36 **
27	5.98 ± 0.23 ***
28	5.49 ± 0.41 ****

* *p* < 0.05 vs. No. 5, ** *p* < 0.05 vs. No. 8, *** *p* < 0.05 vs. No. 11, **** *p* < 0.05 vs. No. 17; *n* = 3. The composition of the microcapsules according to their number is presented in Table 3.

**Table 7:** Droplet size of essential oil in the emulsions.

No. of Emulsion	Droplet Size, μm
1	6.68 ± 1.49
2	7.68 ± 2.55 *
3	8.36 ± 1.98 **
4	8.37 ± 2.30 ***
5	6.08 ± 1.58
6	6.22 ± 1.43
7	6.54 ± 1.75 *
8	6.53 ± 1.89
9	7.32 ± 1.41 *
11	6.72 ± 2.09
12	7.03 ± 2.13 *
13	7.74 ± 2.50 **
14	8.44 ± 2.42 ***

* *p* > 0.05 vs. emulsion containing 0.1% essential oil, ** *p* > 0.05 vs. emulsion containing 0.2% essential oil, *** *p* > 0.05 vs. emulsion, containing 0.3% essential oil; *n* = 3. Composition of emulsions according to their number is presented in the Table 2.

## Data Availability

All data is contained within article.

## Supplementary Materials

The following supporting information can be downloaded at: https://www.mdpi.com/article/10.3390/pharmaceutics14061259/s1, Table S1: Composition of *Boswellia serrata* essential oil obtained by GC-MS.
