# Supplementary material for: Influence of Technological Factors on the Quality of Chitosan Microcapsules with Boswellia serata L. Essential Oil
Source: Pharmaceutics. 2022 Jun 13;14(6):1259. doi: 10.3390/pharmaceutics14061259 (PMC9227605; doi:10.3390/pharmaceutics14061259)
Supplement: Supplementary file 1 [file pharmaceutics-14-01259-s001.zip › pharmaceutics-1724133-supplementary.pdf]

Table S1. Composition of *Boswellia serrata* essential oil obtained by GC-MS

No.	Compounds	Retention time, min	Composition, %
1.	Alpha-phellandrene	8.358	25.41
2.	Tricyclo[3.1.1.03,6]heptane-6-carboxylic acid	8.383	6.00
3.	Alpha-Pinene	8.782	46.90
4.	7-(prop-2-yl)cyclohepta-1,3,5-trie	9.060	0.08
5.	2,2-dimethyl-3-methylene-bicyclo [2.2.1]hep	9.150	0.02
6.	Sabinene	10.600	4.17
7.	1,6-Octadiene, 7-methyl-3-methylene-	11.678	1.42
8.	1,3-Cyclohexadiene, 2-methyl-5-(1-methylet	12.196	0.23
9.	1,3,6-Octatriene, 3,7-dimethyl-, (E)-	12.611	1.78
10.	1,3-Cyclohexadiene, 1-methyl-4-(1-methylet	12.904	0.06
11.	Benzene, methyl(1-methylethyl)-	13.541	1.71
12.	Limonene	13.839	2.73
13.	1,3,6-Octatriene, 3,7-dimethyl-, (E)-	14.444	0.07
14.	Bicyclo[3.1.0]hex-3-en-2-ol, 2-methyl-5-(1-	14.985	0.16
15.	1-isopropyl-4-methyl-1,4-cyclo	15.580	0.17
16.	4-Thujanol	16.117	0.08
17.	1-methyl-4-(1-methylethylidene)	17.402	0.05
18.	2-Oxabicyclo[2.2.2]octane, 1,3,3-trimethyl-	18.139	0.11
19.	1,6-octadien-3-ol, 3,7-dimethyl-	18.504	0.10
20.	1-isopropyl-4-methylbicyclo	19.253	0.34
21.	4,7,7-trimethylbicyclo[4.1.0]hept-	19.715	0.12
22.	4(10)-Thujen-3-ol, stereoisomer	20.974	0.10
23.	2-Pinen-4-ol	21.255	0.06
24.	4(10)-Thujen-3-ol, stereoisomer	23.228	0.23
25.	3-Cyclohexen-1-ol, 4-methyl-1-(1-methyleth	23.545	1.00
26.	Benzenemethanol, alpha, alpha,4-trimethyl	24.309	0.04
27.	3-Cyclohexene-1-methanol, alpha, alpha,4-	24.536	0.07
28.	Anisole	25.120	2.16
29.	Cyclohexanol, 2-methyl-5-(1-methylethenyl)	28.521	0.03
30.	linalyl acetate	28.911	0.05
31.	1,2,3-Trimethyl-cyclopent-2-enecarboxaldeh	29.727	0.03
32.	3-Cyclohexene-1-methanol, alpha, alpha,4-	34.808	0.06
33.	Copaene	36.227	0.11
34.	Beta-bourbonene	36.849	0.75
35.	Bicyclo[7.2.0]undec-4-ene, 4,11,11-trimethyl	37.554	0.09
36.	Benzene, 1,2-dimethoxy-4-(2-propenyl)-	38.690	0.32
37.	1,6-Cyclodecadiene, 1-methyl-5-methylene-8	38.844	0.06
38.	1,6-Cyclodecadiene, 1-methyl-5-methylene-8	39.480	0.06
39.	2-Norpinene, 2,6-dimethyl-6-(4-methyl-3-pe	40.102	0.06
40.	(S,1Z,6Z)-8-Isopropyl-1-methyl-5-methylene	40.404	0.05
41.	8-isopropyl-1-methyl-5-methyle	42.697	0.21
42.	Bicyclo[7.2.0]undec-4-ene, 4,11,11-trimethyl	42.827	0.18
43.	Kessane	45.404	0.51
44.	Cyclohexanemethanol, 4-ethenyl-alpha	46.905	0.03
45.	Benzene, 1,2,3-trimethoxy-5-(2-propenyl)-	47.793	0.23

46.	Alpha-Phellandrene, dimer	60.074	0.39
47.	Hexadecanal	61.559	0.05
48.	Camphorene <m->	69.737	0.09
49.	9-Octadecenal	70.150	0.03
50.	24-Norursa-3,12-diene	88.218	1.22
